# A Comparison of Methods for Assessing the Antioxidant Expression in *Posidonia oceanica* (L.) Delile

**DOI:** 10.3390/molecules30081828

**Published:** 2025-04-18

**Authors:** Debora Fontanini, Fabio Bulleri, Chiara Ravaglioli, Antonella Capocchi

**Affiliations:** 1 Department of Biology, University of Pisa, Via L. Ghini 13, 56126 Pisa, Italy; chiara.ravaglioli@biologia.unipi.it (C.R.); antonella.capocchi@unipi.it (A.C.); 2 MARinePHARMA Center, University of Pisa, Via Bonanno Pisano 6, 56126 Pisa, Italy

**Keywords:** antioxidant, polyphenol, flavonoid, proanthocyanidin, tannin, QUENCHER

## Abstract

Non-enzymatic antioxidants, such as polyphenols, can counteract free radicals and other potentially toxic oxidants produced by marine plants exposed to stress. In this study, we assessed different methods for measuring antioxidant capacities and condensed tannins in the seagrass *Posidonia oceanica* (L.) Delile. Two polyphenol extraction methods, direct and sequential, were compared to determine their efficiencies. Condensed tannins were assayed directly on leaf flour using a modified HCl-butanol-acetone-iron reagent method. Total antioxidant capacities were assayed with the ABTS, CUPRAC, and ORAC methods, both on extracts and on powdered samples (QUENCHER). The direct assays showed higher sensitivity compared to their in-solution counterparts. Our results indicate that in-depth measurement of antioxidant compounds and capacities can be achieved by direct assays on *P. oceanica* powder samples, and these data can be used to assess changes in the plant biochemistry due to the exposure to varying biotic and abiotic conditions.

## 1. Introduction

*Posidonia oceanica* (L.) Delile is a marine plant belonging to the monogeneric Posidoniaceae family, endemic to the Mediterranean and characterized by slow growth and a long lifespan. It forms extensive subtidal meadows across a depth range that extends from 0 to 40/50 m, sustaining coastal biodiversity and ecosystem functioning [1]. The health and distribution of *P. oceanica* meadows, despite being listed as habitats of Community interest [2], are threatened by multiple anthropogenic stressors [3,4,5]. Eutrophication, pollution, coastal erosion, and alien species introduction, as well as climate changes, are among the major threats to *P. oceanica* [6]. Since 1960, there has been an overall decline of 13–50% in the extent of *P. oceanica* [7]; however, possibly due to the implementation of conservation strategies, this declining trend has significantly slowed down [8]. Knowledge of defensive strategies and stress responses that *P. oceanica* activates to withstand hostile environmental conditions is pivotal for its conservation. In addition to anthropogenic stressors, abiotic (e.g., light and temperature) and biotic factors (e.g., herbivory) can also negatively impact plant physiology and growth [9,10,11,12].

Reactive oxygen species (ROS) are produced by plants as stress signaling molecules and by-products released from disrupted metabolic pathways [13]. If the plant scavenging systems fail to counteract those compounds, ROS can cause oxidative damage to membranes, proteins, and nucleic acids, ultimately triggering cell death. For instance, in case of high solar radiation, stress-related chloroplast ROS are overproduced during photosynthesis light reactions at the PSII reaction center, PSI electron transport chains, and LHCII, and under CO_2_-limiting conditions, when ATP synthesis is impaired [14]. ROS damage to the photosynthetic apparatus, which is constantly exposed to light intensity fluctuations, is prevented by a network of antioxidants, such as carotenoids. These compounds scavenge ROS from the antenna complexes and the PSII reaction center, absorbing excess energy from the chlorophyll triplet, thus preventing the formation of the O_2_ excited singlet, which, in turn, causes generation of superoxide anion, hydrogen peroxide, and hydroxyl radical [14]. Further damage to the photosynthetic system is caused by the photooxidation of the chlorophylls and their bleaching [15]. The cell/tissue damage that might result from ROS produced under low-to-moderate light stress is prevented or mitigated by the homeostatic role played by the plant’s scavenging enzymatic (e.g., superoxide dismutase, catalase, ascorbate peroxidase, and glutathione reductase) and non-enzymatic systems. Phenolic compounds, including phenolic acids, flavonoids, stilbenes, coumarins, tannins, and lignans, are important non-enzymatic ROS scavengers. These molecules, both as simple phenolics or polyphenols, play important antioxidant functions as hydrogen or electron donors on account of their aromatic structure and the hydroxyl substituents on their benzene rings [16,17].

Several authors have shown the correlation between polyphenols and biotic/abiotic stress in seagrasses, supporting their role as antioxidants under stressful conditions [18,19,20,21,22,23,24] and their possible use as stress biomarkers. Thus, measuring the antioxidant capacity and characterizing the antioxidant compounds in plant tissues can provide useful elements for assessing plant responses under different abiotic and biotic conditions, such as epiphytic coverage [21], herbivore grazing [25], salinity [20], UV filters [23], light availability and temperature [26], and ocean acidification [27].

Due to the fundamental ecological role of *Posidonia oceanica* meadows, it is critical to be able to properly assess its ability to adapt and thrive under different environmental conditions. Antioxidant compounds and their expression modulation upon exposure to stressful agents are a significant part of plants strategies for coping with varying environmental conditions; therefore, a thorough analysis of those factors can foster our understanding of the effects of adverse conditions on *P. oceanica* meadow health and of biochemical mechanisms underlying its survival strategy. Here, we implemented novel methods for the exhaustive extraction and quantification of antioxidant compounds, such as polyphenols, flavonoids, and tannins, in *Posidonia oceanica*.

## 2. Results

### 2.1. Polyphenols Extraction and Quantification

Polyphenol antioxidant compounds were extracted based on two methods and then assayed for both phenolic compounds and flavonoids. The first method, i.e., direct extraction, which allows for a rapid survey of the methanol-soluble phenolics, is based on extracting the leaf powder with 50% methanol. Instead, the second method, i.e., sequential extraction, separates free and bound polyphenols sequentially; a first step extracts the free polyphenols from the leaf powder with 80% methanol, and a second step extracts the bound polyphenols from the residue via basic hydrolysis followed by acidification and partitioning with diethyl ether/ethyl acetate.

**Figure 1:**
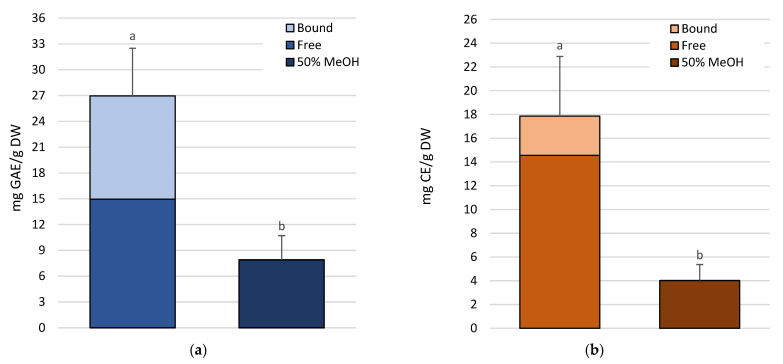
Phenolic compounds (**a**) and flavonoids (**b**) content in *P. oceanica* leaves extracted with the direct (50% MeOH) and sequential (free + bound) methods. GAE, gallic acid equivalents; CE, catechin equivalents; DW, dry weight. Different letters indicate significant differences between extraction methods (*p* < 0.05).

There were significant differences between the two extraction methods in the content of both phenolic and flavonoid compounds (Tables S1 and S2), with the sequential method allowing for a larger amount of polyphenols to be extracted (Figure 1a,b).

Indeed, the ratio between free plus bound and soluble phenols and flavonoids (Figure 1) extracted with 50% methanol averaged 3.4x and 4.4x, respectively. In general, the presence of a higher methanol concentration in the solvent determined a greater extraction of free phenolics and flavonoids; nonetheless, the higher amount of total metabolites extracted with the sequential method was, at least in part, due to the contribution of bound compounds. Those represented, on average, 44.5% and 18.5% of the total phenolics and flavonoids, respectively (Figure 1).

### 2.2. Condensed Tannins (Proanthocyanidins) Quantification

**Figure 2:**
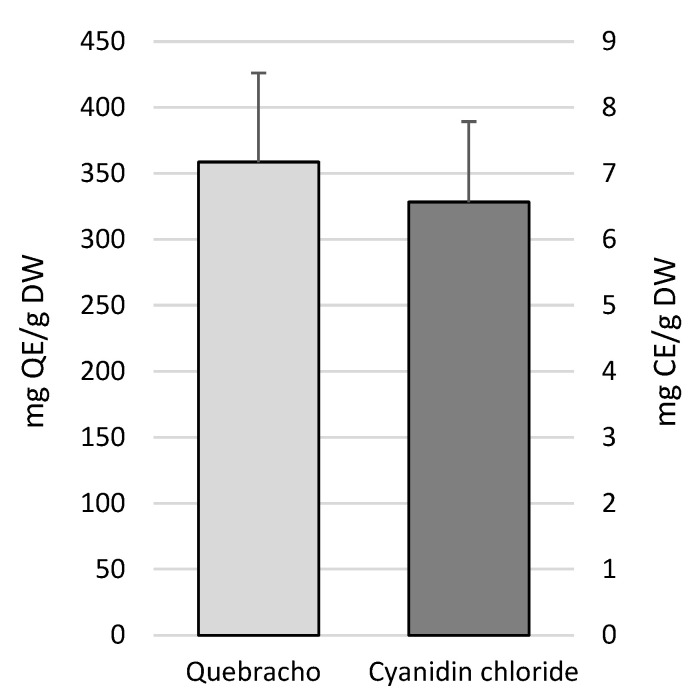
Condensed tannins (proanthocyanidins) content in *P. oceanica* leaves. CE, catechin equivalents; QE, quebracho equivalents; DW, dry weight.

The condensed tannins were quantified based on both cyanidin chloride and quebracho tannin standards. The content of condensed tannins determined by the quebracho standard was larger compared to that quantified with the cyanidin chloride standard (Figure 2). In particular, proanthocyanidin (PA) amounts were within the ranges of 199.32–517.02 ± SD mg quebracho eq/g DW and 3.68–9.43 ± SD mg cyanidin eq/g DW (Figure 2).

### 2.3. QUENCHER (QUick Easy New CHEap Reproducible) Method for Antioxidant Capacity Assays

**Figure 3:**
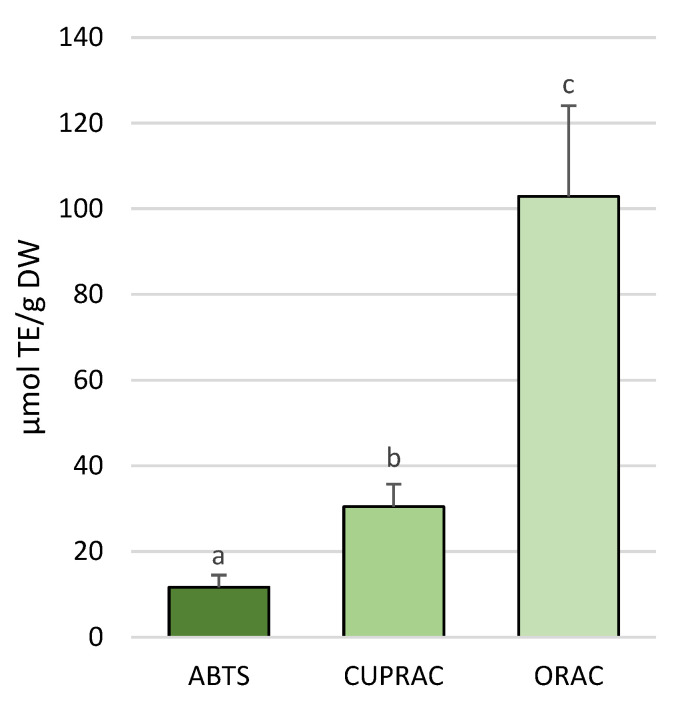
Total antioxidants (TACs) in *P. oceanica* leaves measured by the ABTS, CUPRAC, and ORAC assays with the QUENCHER method. TE, Trolox equivalents; DW, dry weight. Different lowercase letters indicate statistically significant differences between assay methods (*p* < 0.0002).

The total antioxidant capacities (TACs) significantly differed among the three methods (Table S3). In particular, different concentrations were provided by the different methods, with the ABTS (2,2′-azino-bis(3-ethylbenzothiazoline-6-sulfonic acid) assay showing the lowest values, followed by CUPRAC (CUPric ion Reducing Antioxidant Capacity) and ORAC (Oxygen Radical Absorbance Capacity), which resulted in the most sensitive method (Figure 3).

### 2.4. “In-Solution” vs. QUENCHER Total Antioxidant Capacity Assays

**Figure 4:**
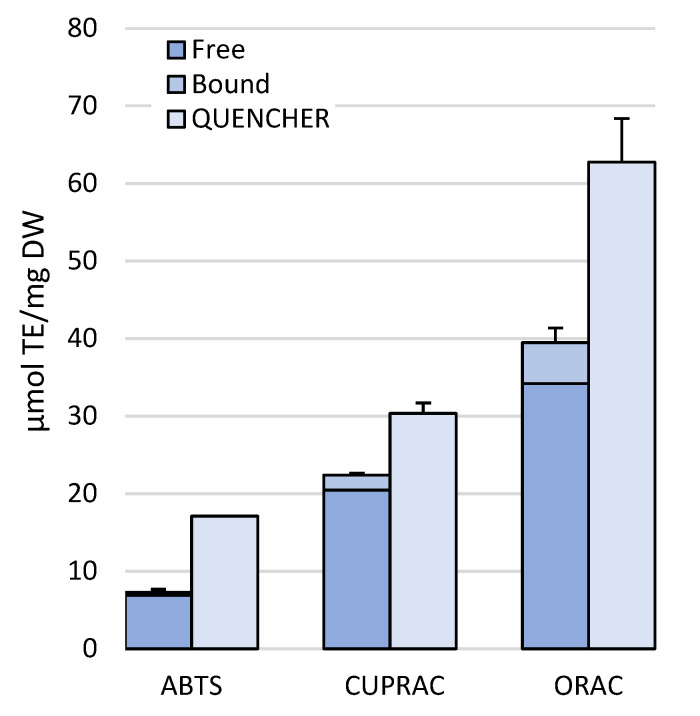
Total antioxidants (TACs) in leaves collected at 20 m depth. Free and bound TACs are shown in the same bar and compared with TACs measured with the QUENCHER. TACs were expressed as μmol TE/g DW. TE, Trolox equivalents; DW, dry weight.

TACs were assessed both on extracts of free and bound polyphenols (in-solution) from leaves collected at 20 m depth and directly onto the leaf powder (QUENCHER) to compare the efficiency of the antioxidant capacity measurement methods on different matrices (Figure 4). The overall trend indicates the ABTS assay as the least efficient method, followed by the CUPRAC and the ORAC. Within each assay method, the direct one was more sensitive to TACs than the in-solution one, with the QUENCHER method detecting on average 56.5%, 26.2%, and 37.1% more TACs than the direct one in the ABTS, CUPRAC, and ORAC assays, respectively (Figure 4).

## 3. Discussion

Several studies have investigated the content of the non-enzymatic antioxidants, such as polyphenols, in *Posidonia oceanica* (L.) Delile under physiological and/or stressful conditions [20,21,28,29,30,31,32,33]. However, little attention has been given to the extraction method aimed at the quantification of total metabolites. Polyphenols are widely recognized as antioxidants involved in plant defense from biotic and abiotic stresses [34,35]. Here, we provide indications of extraction techniques allowing the comprehensive and rapid quantification of antioxidant compounds in *P. oceanica*.

Two different extraction methods to obtain accurate polyphenols measurements were tested and compared. Whereas the first method uses a single extraction step in organic solvent (methanol), the second one involves a more in-depth sequential extraction of free (FP) and bound phenols (BP) [28], as modified by us based on Chu et al. [36] for FP and on Krygier et al. [37] for BP. Unlike the methanol direct extraction, which yields the free (or soluble) phenolic compounds, the second method also allows the extraction of phenolic compounds bound to fibers and proteins, thus ensuring a more complete evaluation of polyphenol content. Polyphenols are often extracted from *P. oceanica* by using organic solvents (methanol or ethanol), and we previously found a 50% methanol solution as suitable for total polyphenol extraction [27]. However, this concentration was less effective than the 80% solution used in the sequential method for a more exhaustive extraction. On the other hand, Astudillo-Pascual et al. [38], investigating stress markers in *P. oceanica* leaves, found better results when using a 50% methanol extractant compared to an 80% solution.

Despite its limitations [39], most of the published studies analyze the phenol content using the Folin–Ciocalteau assay, which reveals colorimetrically the phenolic compounds in a sample and shows high correlation with the sample’s antioxidant potential [40]. In our study, we also measured flavonoids, as a subgroup of polyphenols, to collect some informative data on the mixtures’ chemical nature, albeit at a basic level, as our focus did not include chemical identification.

Comparisons with previous studies are challenging due to differences in extraction methods, units, and standards used, as well as limited methodological or sampling details provided. Furthermore, since polyphenols as secondary metabolites play a role in regulating plant interactions with biotic and abiotic factors, their concentration can vary significantly among meadows exposed to different natural and anthropogenic conditions (e.g., nutrients, seasonality, depth, pollutants, grazers, and epiphytes). Our results are similar to those reported in Kesraoui et al. [28], which also used a sequential extraction to evaluate free and bound polyphenols. In contrast, our bound fractions contained approximately twice the amount of polyphenols. In addition to intrinsically different amounts of polyphenols expressed in response to the local environmental conditions to which the meadows are exposed, this discrepancy could also be attributed to (i) a more exhaustive extraction method involving overnight mixing at 5 °C instead of a 1.5-h sonication/homogenization step and (ii) cold centrifugation instead of filtration, where the filter may have retained some of the extracted compounds. In line with our findings, phenolics in fresh leaves collected at 2–4 m depth and analyzed by HPLC and NMR amounted to 10.66–13.57 mg/g DW as a sum of the prevalent chicoric and caftaric acids [41]. On the other hand, given a 10% extraction yield as reported by Cornara et al. [31], their ethanolic extract contained about 126 μg total polyphenols per gram of extract DW, falling within a range of about 3–7% of the sum of FP and BP that we measured. Also, compared with the direct-methanol extract, extracting with acidified ethanol seems to be less efficient (8.8–53%).

Flavonoids were detected in *P. oceanica* by Cannac et al. [42,43], although at much lower levels compared to the 1.6–6.58 mg CE/g DW found in our samples. However, direct comparison is challenging as flavonols and proanthocyanidins underwent separate extractions, and PAs lacked quantification. In Cannac et al. [42], the authors found significant losses of both compound classes due to chilling and freeze-drying. Conversely, no polyphenol loss was found upon lyophilization by Grignon-Dubois and Rezzonico [41]. In this study, *P. oceanica* leaves, extracted with 50% methanol, contained lower amounts of flavonoids compared to Kesraoui et al. [28], where the same method measured 139.2 mg QE/g extract. This discrepancy was attributed to the lack of specificity of the colorimetric assay used by Kesraoui et al. [28], compared to HPLC analyses. While there is consensus about the scarce specificity of the Folin–Ciocalteu assay due to interference from non-phenolic reducing molecules and other substances (such as reducing sugars, aromatic amines, sulfur dioxide, ascorbic acid, organic acids, and ferrous iron) [44], the aluminum chloride assay for flavonoid quantification in matrices is more specific. It reacts with flavonoids, and when NaNO_2_ is used in the assay, the main drawback is the use of an external standard for the quantification [45]. This is often preferred over an internal flavonoid standard, which requires a complex and time-consuming purification process. In the absence of a shared protocol for *P. oceanica* flavonoid quantification, we selected catechin as the standard due to its widespread use, which allows us to relate data from different studies.

Tannins, a group of structurally complex plant polyphenols, are characterized by high antioxidant power due to their numerous hydroxyl groups. They protect the plant from various abiotic stresses, even though they are particularly effective as feeding deterrents against herbivores thanks to their high protein-binding capacity. They exist in two forms: hydrolyzable and condensed (proanthocyanidins, PAs). While hydrolyzable tannins are not found in seagrasses or in terrestrial monocots [46], condensed tannins are widespread throughout the plant kingdom. In *P. oceanica* they are sequestered, along with other phenolic compounds, in the tannin cells found both in the leaves and rhizomes [47]. As a methodological approach, we measured PAs directly on the leaf powder instead of an extract, to measure the HMW tannins and those bound to fibers and avoid underestimation of the compounds [48]. In fact, the butanol-HCl method on which the assay is based, involving the oxidative depolymerization of the PAs and conversion of the monomers into anthocyanidins, allows for direct quantification of both the soluble and insoluble PA fractions, allowing for in-depth quantification [49]. The variability in composition and polymerization grade of proanthocyanidins, the extraction and assay methods, as well as the choices of quantification standards, makes direct comparisons with literature data challenging. To the best of our knowledge, no literature data detail the chemical composition of *P. oceanica* condensed tannins. However, this plant contains large amounts of catechin amongst its polyphenols, constituting 84.8% of a hydrophilic ethanol extract from leaves [29]. Therefore, it seems reasonable to infer catechin as a component of *P. oceanica* proanthocyanidins, which produces cyanidin upon oxidative hydrolysis in HBAI (HCl-butanol-acetone-iron) reagent [50], making it a suitable standard for its quantitative assessment. We also employed the homemade condensed tannin standard quebracho [51] as a natural quantifiable reference synthesized from catechin as a starter unit [52]. Even though quebracho is resistant to oxidative depolymerization in HBAI, yielding low color and overestimating tannins, it seemed meaningful to standardize PA measurements with it to more easily compare our data with those in the literature expressed as catechin equivalents. Being the constituent anthocyanidins released upon PA hydrolysis, rather than catechin, commonly used in the literature but which would not adequately assess the products of our assay, we used commercial cyanidin as a reference standard for quantification. Moreover, catechin is not considered an appropriate standard, based on its inability to react with acid butanol to yield an anthocyanidin [53]. Insufficient data are available on PA quantification in *P. oceanica*. For instance, Kesraoui et al. [28], using the vanillin assay, measured around 14 mg CE/g DW in leaf samples, which is a little higher than our tannin-richest sample. In *P. australis*, Torbatinejad et al. [54] found 17.4 g/kg DM tannins in leaves collected at 1 m depth using the vanillin/HCl assay method; however, these authors did not report details of the standard used for quantifications, and, assuming it was catechin, we found about double their amounts in our samples.

The QUENCHER (QUick Easy New CHEap Reproducible) approach, although originally developed to directly measure TAC (Total Antioxidant Capacity) in foods, could be easily applied to the *Posidonia oceanica* leaf powder. Antioxidants, occurring as a mixture of soluble, insoluble, and matrix-bound forms, are part of a redox dynamic system where mutual interactions can lead to synergistic, antagonistic, or additive effects [55]. Conventional extraction methods, which measure the TAC of only the extractable fractions, often overlook the interactions between all antioxidant forms. In contrast, the QUENCHER method, which simultaneously assesses all antioxidant fractions in a sample [56], accounts for the antioxidant capacity arising from all possible interactions among the molecules in the analyzed matrix [55], as it may occur when the analyses are conducted on extracts. Indeed, hardly any extraction solvent can solubilize all the antioxidant molecules present in a solid sample, particularly the bound insoluble ones. The power of the QUENCHER approach lies in the assay of choice being performed by adding the radical solution directly to a finely ground sample (in our study, particle size of 104 μm) so that both free and bound antioxidants can interact with it in a liquid–liquid or solid–liquid interface, respectively.

The ORAC method, which relies on fluorescence emission, demonstrated the highest antioxidant capacity. The CUPRAC (CUPric ion Reducing Antioxidant Capacity) and ABTS (2,2′-azino-bis(3-ethylbenzothiazoline-6-sulfonic acid)) assays measured in the ranges of 26.5–33.7% and 7.4–14% of the ORAC (Oxygen Radical Absorbance Capacity) values, respectively. The use of three different assay methods was justified by the potential mechanistic heterogeneity of the antioxidant molecules present in the tissue sample, ensuring a more comprehensive measurement. While the ORAC assay evaluates antioxidant capacity based on the hydrogen atom transfer (HAT) mechanism, CUPRAC and ABTS are based on ET (electron transfer) mode, although some authors consider the ABTS assay as a mixed mode, evaluating both HAT and ET mechanisms [57]; therefore, with our approach, we meant to assess the antioxidants targeted against a range of free radicals, singlet oxygen, transition metal ions, and reactive nitrogen species. Comparing the sum of the TAC measured on the sample collected at −20 m depth by the in-solution assay (free plus bound extracts) with the values obtained using the direct method on the powders, all the assay tested proved more efficient if executed with the QUENCHER method. We speculate that the multiple steps required for a thorough extraction may have not only led to some losses but also failed to release all the antioxidants. In contrast, the QUENCHER approach enabled us to measure a higher amount of bound antioxidant activity and assess potential additive or synergistic effects. Notably, despite being the least performing assay overall, the ABTS assay was the most effective in the QUENCHER modality, detecting approximately 57% more antioxidant capacity than the in-solution ABTS assay, compared to the increases observed with QUENCHER-CUPRAC and ORAC (26% and 37% more, respectively). Likely, when the ABTS assay is performed in the QUENCHER mode, the sample ET antioxidants are more easily contacted by the ABTS radical. On the other hand, the CUPRAC and ORAC assays had already reached a high level of detection in the in-solution assay, so that the improvement by the direct assay was less pronounced.

Unfortunately, there is a lack of literature on TAC measured with the same assays we used, as most of the works apply the DPPH (2,2-diphenyl-1-picrylhydrazyl) and/or FRAP (Ferric Reducing Antioxidant Power) methods [20] and focus on compounds of clinical interest [29,30,31,32,33]. To the best of our knowledge, the only exception is the work of Costa et al. [21], which correlated the ABTS and ORAC assay values with the stress induced by epiphytes in *P. oceanica* leaves collected at a depth of 4–5 m. The antioxidant compounds, extracted with HCl from powdered samples, exhibited values ranging approximately between 2.5–6 and 5.5–8.5 μmol TE/g DW for ABTS and ORAC, respectively. In contrast, our samples, also collected at 5 m and tested using the direct assays, exhibited values that were on average 3 and 15 times higher, respectively, supporting the efficacy of our approach. Although for comparison purposes it may not be needed to perform an exhaustive measurement of the total antioxidant capacity, different classes of antioxidant molecules may perform differently and may interact with each other in different ways so as to escape the traditional extraction and detection techniques. To avoid possible extraction-dependent alteration of the antioxidants’ quality and amount, we applied the QUENCHER method, and the results obtained indicated this strategy as suitable for application to non-food samples.

## 4. Materials and Methods

### 4.1. Study Site

*Posidonia oceanica* plants were collected from a well-preserved meadow at Capraia Island (43°03′08.6″ N; 9°50′17.4″ E) in the Ligurian Sea. In order to encompass a broad gradient of biotic and abiotic conditions, five shoots were randomly collected by divers at different depths (5 m, 10 m, 15 m, 20 m) in September 2018. Plant material was kept in dark coolers and rapidly transported to the laboratory and preserved at −30 °C until analyses.

### 4.2. Plant Material

The second youngest leaf was collected from each of five randomly chosen *Posidonia oceanica* shoots at 5, 10, 15, and 20 m depths. Thawed leaves were scraped with a razor blade to remove epiphytes, briefly rinsed with distilled water, wiped dry with a paper cloth, and lyophilized. The dried leaves were grouped together based on the collection depths and ground for 15 s in a steel ball mill (Retsch GmbH & Co., KG, Haan, Germany) cooled with dry ice. The powders obtained were passed through a 160 mesh (104 μm) with a mechanical sieve (Retsch GmbH & Co., KG, Haan, Germany) and then stored at −30 °C until analyzed.

### 4.3. Phenolic Compounds Extraction

Two different methods were employed for the extraction of total phenolics. In the first one, 5 mg of leaf powder were extracted with 1 mL of cold, 50% methanol in water on a magnetic stirrer for about 16 h at 5 °C. The solution was then centrifuged for 15 min at 13,500× *g* and 5 °C, and the supernatant constituted the total phenolics extract (TPE) [27]. Each extraction had three technical replicates.

The second method was our modification of Kesraoui et al. [28], based on Chu et al. [36] and Krygier et al. [37]. A 5 mg aliquot of leaf powder was extracted on a magnetic stirrer for 18 h in 1 mL of cold, 80% methanol in water at 5 °C. The extract was centrifuged for 10 min at 13,500× *g* and 5 °C. The supernatant was further treated to obtain the free phenolics (free phenolics extract, FPE), whereas the pellet was further extracted for the bound phenolics (bound phenolics extract, BPE).

Free phenolics. After evaporating the supernatant’s methanol in a rotary evaporator (Laborota 4000 efficient, Heidolph Instruments GmbH & CO., Schwabach, Germany), the water residue (FPE) was brought to 200 μL with distilled water and used for the free phenols and flavonoids quantification (see below).

Bound phenolics. The pellet was fully dried under an N_2_ stream and hydrolyzed for 4 h at room temperature, in the dark, with 150 μL of 4 N NaOH. The hydrolysate was then acidified to pH 2.0 with 1 N and 6 N HCl; to obtain the desired final pH, tests were made with a larger 4 N NaOH sample, and then the HCl volume needed to adjust the pH to 2.0 was normalized to the 150 μL sample volume. The acidified sample was extracted five times by partitioning with an equal volume of diethyl ether/ethyl acetate (*v*/*v*, 1:1) for 5 min; the upper, organic phases were pooled and dried under an N_2_ stream. The powder obtained was suspended in 200 μL of distilled water (BPE) and used for the quantification of bound phenols and flavonoids (see Section 4.5). Each extraction was repeated three times (technical replicates).

### 4.4. Phenol and Flavonoid Assays

Phenols and flavonoids were measured both in the methanolic TPE and in the FPE and BPE.

The phenols assay was based on the Folin–Ciocalteu method [58]. Briefly, the extract volume (100 μL TPE; 50 μL FPE and BPE) was brought to 625 μL with distilled water and added with 125 μL of Folin–Ciocalteu reagent (Sigma-Aldrich, Milan, Italy). After vortexing, the sample was kept in the dark for 6 min and added with 1250 μL of 7% Na_2_CO_3_ and 1000 μL of distilled water. The mixture was vortexed and kept in the dark for 90 min. The colored reaction product was read at 760 nm against a blank reaction mixture where water replaced the phenolic extract. The amount of phenols was estimated from a calibration curve made with gallic acid (Sigma-Aldrich, Milan, Italy), and the results were expressed as mmol GAE/g DW.

The flavonoid assay was based on the colorimetric method described by Dewanto et al. [59]. The extract volume (200 μL TPE; 145 μL FPE and BPE) was brought to 1500 μL with distilled water and added with 75 μL of a 5% NaNO_2_ solution. The mixture was vortexed and kept in the dark for 6 min; afterwards, 150 μL of a 10% AlCl_3_·6H_2_O solution were added. After vortexing and standing for another 5 min in the dark, 500 μL of 1 M NaOH and 275 μL of distilled water were added. The solution was vortexed again, and its absorbance was measured immediately at 510 nm against a blank where the sample was replaced with an equal volume of distilled water. The amount of flavonoids was estimated from a calibration curve made with catechin (Sigma-Aldrich, Milan, Italy), and the results were expressed as mmol CE/g DW.

Each extract was assayed in three technical replicates (total of 9 reps/sample); data were reported as means ± SD of nine replicates.

### 4.5. Proanthocyanidins (Condensed Tannins) Quantification

Proanthocyanidins were quantified based on our modification of the direct method by Grabber and Zeller [48] using the HCl-butanol-acetone-iron reagent (HBAI) to release the B-linked anthocyanidin. The fresh reagent (15 mL) was pre-pared daily by dissolving 0.0225 g of ammonium iron (III) sulfate dodecahydrate (Sigma-Aldrich, Milan, Italy) in 450 μL of distilled water (3% final) and 750 μL of 37% HCl (5% final), and then adding 6.3 mL of n-butanol (42% final) and 7.5 mL of acetone (50% final). Powder aliquots of 2 mg were reacted with 2 mL of HBAI reagent in screw-capped Pyrex tubes placed in a 70 °C water bath for 3 h and vortexed every 15 min. The tubes were then left to cool down at room temperature for 30 min; a 1.5 mL aliquot of extract was quickly centrifuged, and its absorbance was measured spectrophotometrically at 550 nm against a blank made with the HBAI reagent. Each extraction/assay had five technical replicates.

Proanthocyanidin quantification was based on a calibration curve made with the homemade tannin standard quebracho, purified as described in Brown et al. [51], from quebracho powder extracted from the hardwood tree *Schinopsis lorentzii* (Griseb.) Engl. (kindly provided by Prof. Giulio Petroni). The purified standard was dissolved in water (5 mg/mL) to be subjected to the HBAI assay to free the constituent anthocyanidins. All points had five replicates. Calibration curves were also made with the commercially available anthocyanin cyanidin chloride (Extrasynthese, Genay, France), in the range 0.000883–0.0125 μg/μL, with five technical replicates each.

### 4.6. Total Antioxidant Capacity—QUENCHER (QUick Easy New CHEap Reproducible) (Direct) Method

Total antioxidant capacities (TAC) of the *P. oceanica* leaf samples were measured either directly on the powders (QUENCHER method) or on the free and bound phenolics extracted from leaves collected at −20 m by the ABTS (2,2′-azino-bis(3-ethylbenzothiazoline-6-sulfonic acid)), CUPRAC (CUPric ion Reducing Antioxidant Capacity), and ORAC (Oxygen Radical Absorbance Capacity) methods.

#### 4.6.1. QUENCHER-ABTS

The assay was performed based on our modification of the method by Re et al. [60], using a 160-mesh powder. Briefly, the ABTS radical cation was generated by reacting 25 mL of a 7 mM ABTS (Sigma-Aldrich, Milan, Italy) solution in water with 88 μL of a 0.14 M solution of potassium persulfate for 16 h in the dark at room temperature and then diluting it to 1:83.3 with 50% ethanol to give a solution with an absorbance of 0.7 at 734 nm [61]. Leaf powder aliquots of 1 mg were mixed directly with 12.5 mL of ABTS^+^ on a magnetic stirrer, in the dark, for 6 min. Afterwards, the reaction mixture was quickly centrifuged to pellet the powder, and its absorbance at 734 nm was read against a blank made with ABTS^+^. All assays were made in 9–10 technical replicates.

TAC was estimated based on the ABTS^+^ discoloration % by Trolox (Sigma-Aldrich, Milan, Italy), with a standard curve made in the range 1 µM–20 µM and obtained by mixing 5 µL of Trolox in 50% ethanol (100 µM–20 mM) with 595 µL of ABTS^+^. After incubating for 6 min in the dark, the absorbance was read at 734 nm, and the ABTS^+^ discoloration % was calculated as 100 − (OD0 min/OD6 min) and plotted vs. Trolox concentration. Each point of the standard curve was the average of 5 replicates. TAC was expressed as µmol TE/mg DW.

#### 4.6.2. QUENCHER-CUPRAC

This method was based on our modification of the methods by Tufan et al. [62] and Apak et al. [63]. The CUPRAC reagent was prepared by mixing equal amounts of 10 mM Cu(II) in water, freshly prepared 1 M ammonium acetate, and 7.5 mM neocuproine (Sigma-Aldrich, Milan, Italy) in 96% ethanol. The reagent was diluted 1:5 in water. Leaves powder aliquots (160-mesh, 104 μm) of 1 mg were mixed with 4.1 mL of CUPRAC diluted reagent on a magnetic stirrer for 30 min in the dark. The samples were then centrifuged for 5 min at 13,500 rpm, and their absorbances were read at 450 nm against a blank made by CUPRAC reagent. All assays had three technical replicates. TAC was estimated based on a Trolox calibration curve made in the range of 4.88 µM–78.08 µM and obtained by diluting 4–64 µL of 5 mM Trolox in 96% ethanol with water to 1.1 mL final volume. After adding 3 mL of CUPRAC reagent, the reaction was incubated for 30 min in the dark, and the absorbance was read at 450 nm. Each concentration was measured with 3 technical replicates. TAC was expressed as µmol TE/mg DW.

#### 4.6.3. QUENCHER-ORAC

The assay was modified from the protocols of Huang et al. [64], Amigo-Benavent et al. [65], and Ou et al. [66]. Fluorescein sodium salt (Sigma-Aldrich, Milan, Italy) stock solution (0.611 mM in 75 mM K-phosphate buffer, pH 7.0), stored at −80 °C, was diluted to a 52.5 nM working solution in phosphate buffer and kept on ice. A 153 mM AAPH [2,2′-Azobis(2-methylpropionamidine) dihydrochloride] (Sigma-Aldrich, Milan, Italy) solution in the phosphate buffer was freshly made every day. An opaque tube was used as an assay vessel. The assay tube, containing 0.3 mg of leaf powder and a magnetic stir bar, was placed in a water bath (37 °C) positioned above a magnetic stirrer (speed, 500 rpm). Then, 1500 μL of cold fluorescein working solution were poured in the tube and mixed with 250 μL of phosphate buffer, allowed to equilibrate to 37 °C, and mixed with 250 μL of cold AAPH solution. After 30 s, a 200 μL aliquot of the reaction mixture was pulled, quickly spun (10 s) in a sealed microfuge tube, and its fluorescence was immediately read (λexc = 485 nm, λem = 515 nm) with an RF-6000 spectrofluorophotometer (Shimadzu, Milan, Italy); this reading was set as T0. The mixture was then returned to the tube, and the operation was repeated every 3 min for 90 min. A blank was also run to measure fluorescence decay in the absence of antioxidants. For each assay, a normalized fluorescence decay curve was plotted and after subtracting the blank, the area under the curve was calculated (AUC). ORAC values were expressed as µmol TE/mg DW, based on a Trolox calibration curve established with four Trolox concentrations (3.125, 6.25, 12.5, and 25 µM). All measurements were conducted in triplicate.

### 4.7. Total Antioxidant Capacity—“In-Solution” Method

ABTS, CUPRAC, and ORAC antioxidant assays were also performed on liquid samples. The assays were carried out with the same protocols described for the QUENCHER assays, except for the samples used consisting of appropriate volumes (5.2, 15, and 10 µL of extract, respectively, for ABTS, CUPRAC, and ORAC assays) of free and bound antioxidants extracted from sample leaves collected at 20 m depth.

### 4.8. Statistical Analysis

One-factor ANOVAs were used to assess variations among methods for extracting phenolic compounds and flavonoids (direct extraction vs. sequential) and for assessing the total antioxidant capacity (TAC) (ABTS vs. CUPRAC vs. ORAC). Technical replicates within each depth were averaged, yielding four independent replicates that were used in ANOVAs. Heterogeneity of variances was checked using Cochran’s tests, and data were square-root or log transformed when necessary. The SNK test was used for ranking the means of the different methods assessing total antioxidant capacity.

Variations between methods for extracting tannins (quebracho vs. cyanidin chloride) were not assessed statistically since the values they generate are in different units. Likewise, variations of the “in-solution” vs. QUENCHER total antioxidant capacity assays on plants collected only at a depth of 20 m were assessed only visually since there were no biological replicates.

## 5. Conclusions

The aim of our work was to compare different methods for assessing the antioxidant capacity of *P. oceanica* leaves. In doing so, we employed a sequential extraction method to efficiently measure free and bound polyphenols, which are known for their high antioxidant activity, such as phenols and flavonoids. We also quantified *P. oceanica* proanthocyanidins using a method that directly treats the pulverized sample with the assay reagents, thereby avoiding potential sample losses due to the extraction step(s). We have shown the applicability of the QUENCHER direct assay method for TAC measurements, originally designed for food matrix analyses, and shown that QUENCHER-ABTS, -CUPRAC, and -ORAC assays were more efficient than the in-solution versions. Overall, the extraction and assay methodologies employed in our work were found to be optimal for a detailed quantification of antioxidant polyphenols and their antioxidant capacities.

## Data Availability

The raw data supporting the conclusions of this article will be made available by the authors upon reasonable request.

## Supplementary Materials

The following supporting information can be downloaded at: https://www.mdpi.com/article/10.3390/molecules30081828/s1. Table S1. One-factor ANOVA to assess variations in polyphenol amounts extracted with the direct and the sequential extraction methods. Table S2. One-factor ANOVA to assess variations in flavonoid amounts extracted with the direct and the sequential extraction methods. Table S3. One-factor ANOVA to assess variations in total antioxidant activities (TAC) between measurements made with the ABTS, CUPRAC, and ORAC assays.

## Abbreviations

The following abbreviations are used in this manuscript:

ABTS	2,2′-Azino-bis (3-ethylbenzothiazoline-6-sulfonic acid)
CE	Catechin equivalent
CUPRAC	CUPric ion Reducing Antioxidant Capacity
FRAP	Ferric Reducing Antioxidant Power
GAE	Gallic acid equivalent
HBAI	HCl-butanol-acetone-iron
ORAC	Oxygen Radical Absorbance Capacity
PA	Proanthocyanidin
QUENCHER	QUick Easy New CHEap Reproducible
TAC	Total antioxidant capacity
TE	Trolox equivalent
