# Supplementary material for: A Comparison of Methods for Assessing the Antioxidant Expression in Posidonia oceanica (L.) Delile
Source: Molecules. 2025 Apr 18;30(8):1828. doi: 10.3390/molecules30081828 (PMC12029781; doi:10.3390/molecules30081828)
Supplement: Supplementary file 1 [file molecules-30-01828-s001.zip › molecules-3533097-supplementary.pdf]

Table S1. Results of one-factor ANOVA used to assess variations in polyphenol amounts extracted with the direct and the sequential extraction methods.

	Df	Sum Sq	Mean Sq	F value	Pr(>F)
Extraction method[a]	1	726.19	726.19	9.4723	0.02193*
Residuals	6	462.19	77.03		

[a]Direct (50% MeOH soluble) or sequential (free and bound)

Table S2. Results of one-factor ANOVA used to assess variations in flavonoid amounts extracted with the direct and the sequential extraction methods.

	Df	Sum Sq	Mean Sq	F value	Pr(>F)
Extraction method[a]	1	383.10	383.10	7.0655	0.03762*
Residuals	6	325.33	54.22		

[a]Direct (50% MeOH soluble) or sequential (free and bound)

Table S3. Results of one-factor ANOVA used to assess variations in total antioxidant activities (TAC) between measurements made with the ABTS, CUPRAC, and ORAC assays. Data were square-root (x+1) transformed to remove heterogeneity of variances.

	Df	Sum Sq	Mean Sq	F value	Pr(>F)
Assay method[a]	2	90.061	45.031	24.181	0.00024***
Residuals	9	16.760	1.862		

[a]ABTS, CUPRAC, and ORAC
